# CRBA: A Competitive Rate-Based Algorithm Based on Competitive Spiking Neural Networks

**DOI:** 10.3389/fncom.2021.627567

**Published:** 2021-04-22

**Authors:** Paolo G. Cachi, Sebastián Ventura, Krzysztof J. Cios

**Affiliations:** ^1^Department of Computer Science, Virginia Commonwealth University, Richmond, VA, United States; ^2^Department of Computer Science, Universidad de Córdoba, Córdoba, Spain; ^3^Polish Academy of Sciences, Gliwice, Poland

**Keywords:** rate-based algorithm, competitive spiking neural networks, competitive learning, unsupervised image classification, MNIST

## Abstract

In this paper we present a Competitive Rate-Based Algorithm (CRBA) that approximates operation of a Competitive Spiking Neural Network (CSNN). CRBA is based on modeling of the competition between neurons during a sample presentation, which can be reduced to ranking of the neurons based on a dot product operation and the use of a discrete Expectation Maximization algorithm; the latter is equivalent to the spike time-dependent plasticity rule. CRBA's performance is compared with that of CSNN on the MNIST and Fashion-MNIST datasets. The results show that CRBA performs on par with CSNN, while using three orders of magnitude less computational time. Importantly, we show that the weights and firing thresholds learned by CRBA can be used to initialize CSNN's parameters that results in its much more efficient operation.

## 1. Introduction

A Competitive Spiking Neural Network (CSNN), is a two-layer feedforward spiking network with lateral inhibitory connections (Querlioz et al., 2011; Diehl and Cook, 2015; Cachi et al., 2020), that uses spiking neurons with local Konorski/Hebb learning rule to implement a dynamic temporal network that exhibits properties often missing in deep learning models. They are pattern selectivity (spiking neurons learn to detect specific input patterns) (Masquelier and Thorpe, 2007; Nessler et al., 2009; Lobov et al., 2020), short-/long- term memory (spiking neurons use self-regulatory mechanism that processes information in different time scales) (Ermentrout, 1998; Brette and Gerstner, 2005; Pfister and Gerstner, 2006; Zenke et al., 2015), synaptic plasticity (based on local learning first observed by Konorski and then by Hebb) (Konorski, 1948; Hebb, 1949), modularity (spiking neurons operate and learn independently) (Zylberberg et al., 2011; Diehl and Cook, 2015), adaptability, and continuous learning (Brette and Gerstner, 2005; Wysoski et al., 2008).

In spite of these advantages, the performance of CSNN is still modest in comparison with backpropagation networks (Ciregan et al., 2012; Wan et al., 2013; Diehl et al., 2015; Rueckauer et al., 2017). Two main problems limit usage of CSNN: its very slow learning and testing time, and the difficulty of making sense of its dynamic mechanisms. The latter problem is due to the fact that while it is not easy to analyze just one dynamic mechanism, CSNN uses three types of dynamic mechanisms in its operation: the spike generating process, the adaptable firing threshold, and the spike time-dependent plasticity (STDP) learning rule. Importantly, in addition to the difficulty of concurrently tuning the three dynamic mechanisms, CSNN has an order of magnitude more hyper-parameters than backpropagation networks with similar number of neurons.

CSNN's slow learning and testing times are due to the fact that spiking neurons implementation relies on the use of special purpose hardware, such as a neuromorphic processor, that are not yet freely available. Because of this, the current approach of using CSNN is to implement them using time-step simulation on regular computers, which translates into its very high computational cost. For example, the CSNN implementation used in Diehl and Cook (2015) requires more than 12 h to train 400 spiking neurons on MNIST dataset, and around 44 h to train 1,600 spiking neurons (on Intel core i9 computer with 64 GB of RAM). These two facts make the use of CSNN network very limited.

With the aim of facilitating wider use and understanding of CSNN, in this paper we propose a rate-based algorithm equivalent to CSNN but that is much simple and well-suited to be run on regular computers. The proposed algorithm, CRBA, approximates the temporal processing of inputs carried out by CSNN using a 5-step heuristic (see section 2.3) based on modeling of its operation. CRBA's performance is tested on the MNIST and Fashion-MNIST datasets and the experiments show that it reduces computational cost by up to three-orders of magnitude when compared with CSNN and without reducing accuracy. Additionally, we show that parameters learned by CRBA can be used to initialize CSNN's weights and firing thresholds to make its operation much faster.

The technical contributions of this paper are summarized as follows:

- *Derivation*. Modeling of CSNN operation is described in section 2.2. It is based on the assumption that the network's input is constant during a presentation time, *t*, and that STDP rule is accurately approximated by Expectation Maximization (EM) algorithm (Nessler et al., 2009).
- *Model*. CRBA operation, equivalent to CSNN, is formulated in section 2.3. It consists of a 5-step heuristic. First, all neurons are ranked based on their calculated spiking frequency. Second, a number of winner neurons are selected from the ranking result. Third, a number of spikes each winner neuron generates is calculated. Fourth, the winner neurons' weights and firing thresholds are updated. Finally, its weights are normalized.
- *Performance*. The performance of CRBA is tested on the MNIST and Fashion-MNIST datasets. The results show that CRBA performs on par with CSNN, while using three orders of magnitude less computational time.
- *Application*. We show experimentally that the parameters learned by CRBA can be directly used to initialize CSNN's parameters that makes it much more efficient at the cost of a slight reduction of accuracy. This is important as it shows that CRBA can be used for efficient deployment of CSNN on neuromorphic computers, in particular those that do not allow for on-chip learning.

## 2. Methods

### 2.1. Background

We begin with a brief overview of spiking neurons that mimic the spiking nature of biological neurons and then of the CSNN, which is a two-layer feedforward spiking network that uses STDP-like learning rule.

#### 2.1.1. Spiking Neuron Model

**Figure 1:**
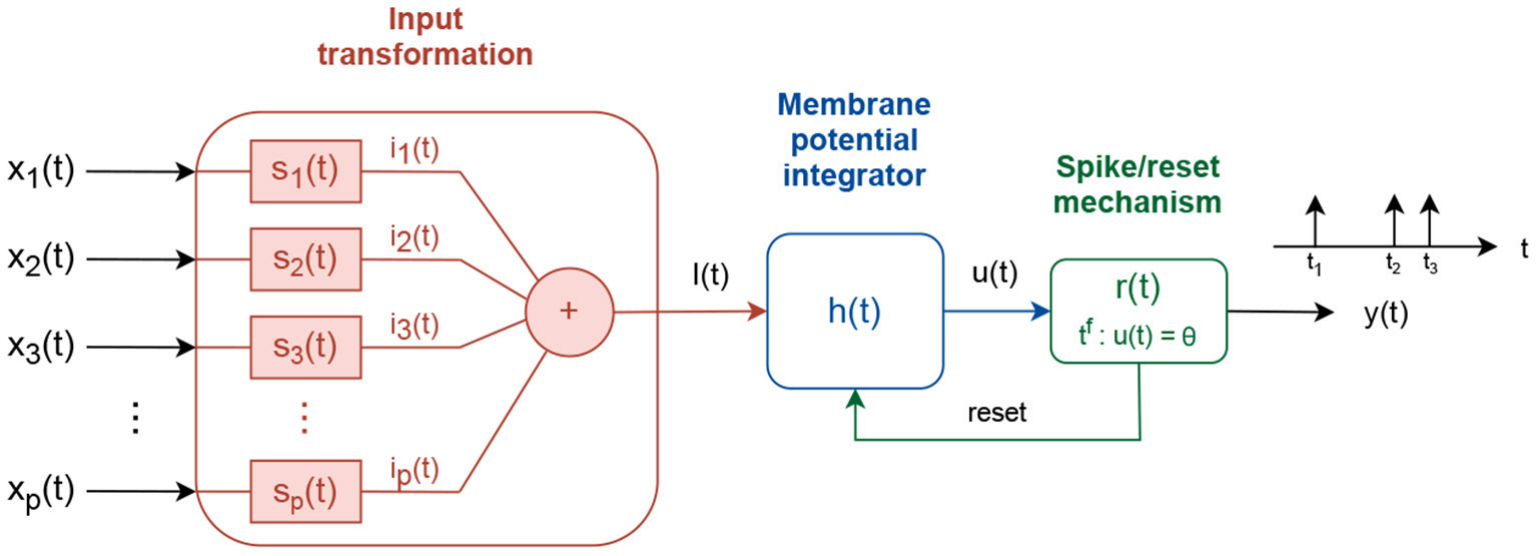
Integrate and fire spiking neuron model.

In contrast to simple neuron models, which calculate nonlinear transformation of real-valued inputs *f* : ℝ^*P*^ → ℝ, spiking neurons integrate time-dependent input signals *f*(*t*) : *X*(*t*) → *y*(*t*), where *X*(*t*) = {*x*_1_(*t*), *x*_2_(*t*)…*x*_*p*_(*t*)} and *y*(*t*) is a train of short pulses called spikes (Koch and Segev, 1998; Kandel et al., 2013; Gerstner et al., 2014). Although different spiking neuron models have been developed, to account for different levels of biological similarity, here we focus on the Integrate and Fire spiking neuron model (Gerstner et al., 2014), shown in Figure 1.

The input signals *x*_*i*_(*t*) are processed via three consecutive transformations to produce an output signal *y*(*t*). First, the input signals are linearly combined into a driving signal *I*(*t*). The linear combination is accomplished using intermediate subsystems, *s*_*i*_(*t*), that scale the input signal, *s*_*i*_(*t*) = *w*_*i*_δ(*t*), where *w*_*i*_ defines the scaling factor and δ(*t*) is the Dirac function, or transform it via an exponentially decay system $s_{i}\left(t\right)=w_{i}e^{t/\tau_{s}}$, where τ_*s*_ is a time decay constant.

Second, the driving signal *I*(*t*) is integrated into an intermediate membrane potential signal *u*(*t*). The membrane potential is built as:

(1) $$\begin{array}{l} \frac{du}{dt}=\frac{f\left(u\right)}{\tau_{f}}+\frac{g\left(u\right)}{\tau_{g}}I\left(t\right) \end{array}$$

where *f*(*u*) and *g*(*u*) are linear/nonlinear functions of the instantaneous membrane potential value *u*, and τ_*f*_ and τ_*g*_ are time decaying constants. *f*(*u*) can be a linear function *f*_1_(*u*), a quadratic *f*_2_(*u*), or an exponential *f*_3_(*u*) function:

(2) $$\begin{array}{l} f_{1}\left(u\right)=-\left(u-u_{r}\right)\; \end{array}$$

(3) $$\begin{array}{l} f_{2}\left(u\right)=-\left(u-u_{r}\right)+\Delta_{T}\exp\frac{\left(u-\vartheta \right)}{\Delta_{T}} \end{array}$$

(4) $$\begin{array}{l} f_{3}\left(u\right)=a_{0}\left(u-u_{r}\right)\left(u-u_{c}\right) \end{array}$$

where *u*_*r*_ represents a resting membrane potential value, Δ_*T*_ the sharpness factor, ϑ the firing threshold variable/constant, and *a*_0_ and *u*_*c*_ are constants with *a*_0_ > 0 and *u*_*c*_ > *u*_*r*_. The term *g*(*u*) is used to couple the driving signal *I*(*t*) into the membrane potential function. It follows direct contribution *g*(*u*) = 1 (Querlioz et al., 2011) or conductance-based contribution *g*(*u*) = (*u*_*i*_ − *u*) (Diehl and Cook, 2015), where *u*_*i*_ is an input reversal potential constant.

Third, the membrane potential *u*(*t*) is pass-through a spike generation mechanism, where spikes are produced every time *t*^*f*^ the membrane potential value crosses, from below, a fixed or an adaptive firing threshold, ϑ. If the firing threshold is adaptive, its value follows an exponential decay process with constant increment of α after each spike:

(5) $$\begin{array}{l} \frac{d\vartheta }{dt}=\frac{\vartheta_{0}-\vartheta }{\tau_{\vartheta_{0}}}+\underset{t^{f}}{\sum }\alpha \delta \left(t-t_{i}^{f}\right) \end{array}$$

where ϑ_0_ is a threshold offset value, and τ_ϑ_0__ a time decay constant. After each spike is generated, a reset signal is used to reset the membrane potential to *u*_*reset*_ and hold it fixed for time *t*_*r*_.

#### 2.1.2. Competitive Spiking Neural Network

**Figure 2:**
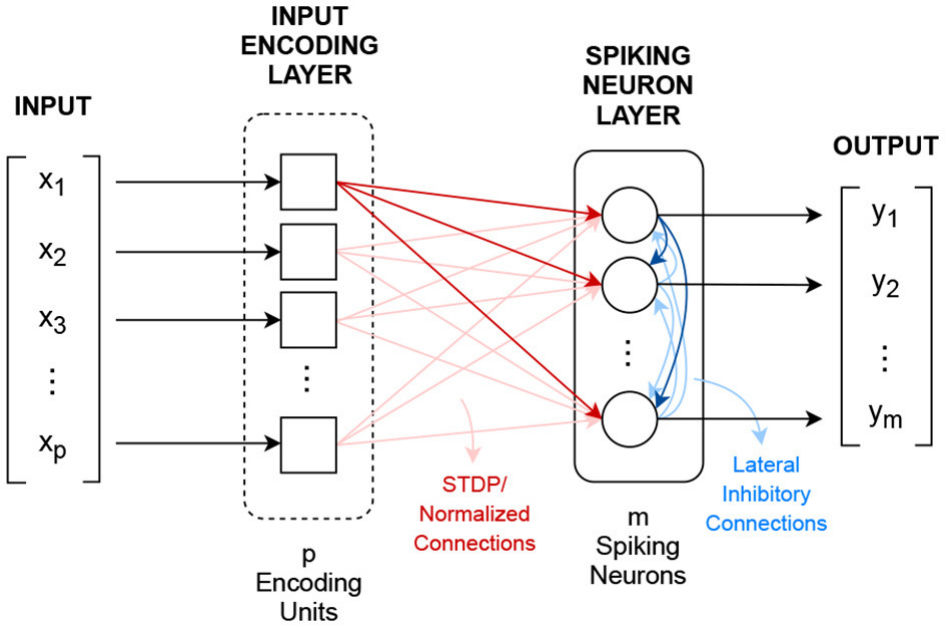
Competitive spiking neural network topology.

A CSNN is a two layer spiking neuron network that implements the winner-takes-all learning mechanism, where spiking neurons compete against each other for the right to represent an input *X* which is closest to their synapse value (Rumelhart and Zipser, 1985; Shin et al., 2010; Querlioz et al., 2011; Diehl and Cook, 2015), where *X* ∈ ℝ^*p*^. Figure 2 shows CSNN's topology.

The first layer, the input encoding layer, transforms the *p*-dimensional input vector *X* into *p* spiking signals *x*_*i*_(*t*). Although different encoding methods can be used, the most common one uses the Poisson's distribution for implementing spike rate encoding.

After encoding, the resulted signals excite, in a fully connected fashion, via normalized learnable synapse connections, *m* threshold-adaptable spiking neurons. These synapses are used as convolutional kernels, *s*_*ij*_(*t*) = *w*_*ij*_δ(*t*), in which the weight values *w*_*ij*_ are adjusted/learned following a normalized version of the STDP learning rule (Gerstner and Kistler, 2002; Morrison et al., 2008), defined in Equation (6).

(6) $$\begin{array}{l} \Delta w_{ij}=\{\begin{array}{cc} \alpha_{+}\exp\left(-\Delta t_{i}/\tau_{+}\right) & \text{if}\Delta t_{ij}>0\\ -\alpha_{-}\exp\left(\Delta t_{i}/\tau_{-}\right) & \text{if}\Delta t_{ij}\leq 0 \end{array} \end{array}$$

The update of the synapse's weight *i* for neuron *j* is calculated based on the exponentially decaying function of time difference between the pre- and post-synaptic spikes Δ*t*_*ij*_. The parameters α_+_ and α_−_ are constant learning rates, and τ_+_ and τ_−_ are exponential decay constants. The normalization is done at the individual neuron level using Equation (7).

(7) $$\begin{array}{l} w_{ij}^{\prime }=w_{ij}\frac{\lambda }{\sum_{i}w_{ij}} \end{array}$$

where $w_{ij}^{\prime }$ is the resulting normalized weight for connection *i* of neuron *j*, and λ is the per-neuron total connection constant (Gerstner and Kistler, 2002; Liang et al., 2018).

In addition to the input connections, the spiking neurons are connected to each other by fixed recurrent inhibitory connections, known as lateral inhibition, with the purpose of feedback regulation (Querlioz et al., 2013; Diehl and Cook, 2015).

CSNN's output is the *m*-dimensional vector *Y* that shows the number of spikes each neuron emits during presentation of a given input.

### 2.2. Rate-Based Model of Competitive Spiking Neural Networks

Here we perform analysis of the temporal operation of CSNN. We start by describing operation of CSNN with only one spiking neuron in its one only spiking layer. Next, we analyze a general case with multiple spiking neurons in its layer. We finish with discussion of the variable firing threshold mechanism and STDP learning rule.

#### 2.2.1. Operation of a Single Neuron

With only one neuron in the spiking layer, this neuron receives excitatory input from time-dependent signals *x*_*i*_(*t*). Using a neuron with linearly decaying term *f*(*u*) = −(*u* − *u*_*r*_) and direct input connection *g*(*u*)/τ_*g*_ = 1, with *p* input signals *x*_*i*_(*t*) through direct synapse connections *s*_*i*_(*t*) = *w*_*i*_δ(*t*), we expand Equation (1) to express change of the neuron's membrane potential *u*(*t*) as:

(8) $$\begin{array}{l} \frac{du\left(t\right)}{dt}=\frac{\left(u_{r}-u\left(t\right)\right)}{\tau_{u}}+\overset{p}{\underset{i=1}{\sum }}w_{i}x_{i}\left(t\right) \end{array}$$

which represents a linear system with impulse response equal to $h\left(t\right)=e^{-t/\tau_{u}}$ (Gerstner et al., 2014). The solution for membrane potential for time *t*, in absence of the firing threshold, resting potential equal to zero and input signal *x*_*i*_(*t*) = 0 for *t* < 0, is found by convolution of the driving signal, $I\left(t\right)=\overset{p}{\underset{i=1}{\sum }}w_{i}x_{i}\left(t\right)$, and the neuron's impulse response, *h*(*t*), expressed by *u*(*t*) = *I*(*t*) * *h*(*t*). This is solved as:

(9) $$\begin{array}{l} u\left(t\right)=I\left(t\right)*h\left(t\right)=\overset{p}{\underset{i=1}{\sum }}w_{i}\int_{0}^{t}e^{-s/\tau_{u}}x_{i}\left(t-s\right)ds \end{array}$$

We can approximate a solution for Equation (9) by considering the input driven signal to be a spiking train signal with constant frequency rate *f*_*i*_ = β*x*_*i*_ (instead of the Poisson distribution resulting spiking signal). Then the product $e^{-s/\tau_{u}}x_{i}\left(t-s\right)$ reduces to a exponential decaying function sampled with frequency rate *f*_*i*_ expressed as $e^{-n/f_{i}\tau_{u}}$, for *n* = 0, 1, 2, …, and the integral reduces to summation over the *N* number of spikes during time *t*, where *N* is calculated as *N* = ⌊*tf*_*i*_⌋.

(10) $$\begin{array}{l} u\left(t\right)\approx \overset{p}{\underset{i=1}{\sum }}w_{i}\overset{N-1}{\underset{n=0}{\sum }}e^{-n/f_{i}\tau_{u}} \end{array}$$

Equation (10) can be approximated by expanding the exponential summation $\overset{N-1}{\underset{n=0}{\sum }}e^{-n/f_{i}\tau_{u}}$ to $\left(1-e^{-n/f_{i}\tau_{u}}\right)/\left(1-e^{-n/f_{i}\tau_{u}}\right)$ and by using $1/\left(1-e^{-1/f_{i}\tau_{u}}\right)\approx f_{i}\tau_{u}$, which was found experimentally by analyzing the behavior of $1/\left(1-e^{-1/f_{i}\tau_{u}}\right)$ as *f*_*i*_τ_*u*_ increases.

(11) $$\begin{array}{l} u\left(t\right)\approx \overset{p}{\underset{i=1}{\sum }}w_{i}f_{i}\tau_{u}\left(1-e^{-t/\tau_{u}}\right)=\tau_{u}\beta \left(1-e^{-t/\tau_{u}}\right)\overset{p}{\underset{i=1}{\sum }}w_{i}x_{i} \end{array}$$

The resultant solution is an increasing function, with time constant τ_*u*_, toward a steady state value at $\tau_{u}\beta \overset{p}{\underset{i=1}{\sum }}w_{i}x_{i}$. The summation term represents dot product between the input vector, *X*, and the synapse connection vector, *W*. Thus, the solution for the membrane potential can be written as:

(12) $$\begin{array}{l} u\left(t\right)\approx \tau_{u}\beta \langle X,W\rangle \left(1-e^{-t/\tau_{u}}\right) \end{array}$$

where 〈*X, W*〉 represents dot product between vectors *X* and *W*. With one spiking neuron, and without a firing threshold, the increase of the membrane potential depends on the level of similarity between the input and its current synaptic weights. Note that without a firing threshold, the synaptic weight stays constant during the interval of time *t*. Consequently, the increase in the membrane potential remains constant for the time interval *t*.

If a firing threshold, ϑ, in the range between *u*_*r*_ and τ_*u*_β〈*X, W*〉, is used, the time *t*^*f*^ at which a neuron generates a spike is given by:

(13) $$\begin{array}{l} t^{f}=\tau_{u}\left[\log\left(\tau_{u}\beta \langle x,w\rangle \right)-\log\left(\tau_{u}\beta \langle x,w\rangle -\vartheta \right)\right] \end{array}$$

which still depends on the level of similarity between the vectors *X* and *W*, and the firing threshold value ϑ.

#### 2.2.2. Operation With Multiple Neurons

In the case of *m* neurons in the spiking layer, each neuron receives input not only from the encoded signals, *x*_*i*_(*t*), but also from recurrent connections—outputs of all other spiking neurons *y*_*k*_(*t*). Assuming that the encoded signals are connected through direct excitatory connections, $s_{i}\left(t\right)=w_{i}^{ex}\delta \left(t\right)$, and the recurrent signals through the inhibitory connections, $s_{k}\left(t\right)=-w_{k}^{in}\delta \left(t\right)$, the membrane potential change for neuron *j* is expressed as:

(14) $$\begin{array}{l} \frac{du_{j}\left(t\right)}{dt}=\frac{\left(u_{r}-u_{j}\left(t\right)\right)}{\tau_{u}}+\overset{p}{\underset{i=1}{\sum }}w_{ij}^{ex}x_{i}\left(t\right)-\overset{m}{\underset{k\neq j}{\sum }}w_{kj}^{in}y_{k}\left(t\right) \end{array}$$

This is similar to Equation (8), the difference being that now the driving signal is processed as a linear combination of excitatory and inhibitory terms. As the membrane potential is a linear system, Equation (14) is solved by adding the individual solutions of each contribution term.

(15) $$\begin{array}{l} u_{j}\left(t\right)=u_{j}^{ex}\left(t\right)+u_{j}^{in}\left(t\right) \end{array}$$

where $u_{j}^{ex}\left(t\right)$ is the membrane potential when only the excitatory signals are used, and $u_{j}^{in}\left(t\right)$ for the inhibitory signals. The solution for the excitatory term has been already shown in Equation (12). To solve for the inhibitory term, we use the fact that the recurrent signals, *y*_*k*_(*t*), are spiking signals of the form $\delta \left(t-t_{k}^{f}\right)$, where $t_{k}^{f}$ is the firing time. As each impulse that passes throughout the system *h*(*t*) transforms into $e^{-\left(t-t_{k}^{f}\right)/\tau_{u}}\mu \left(t-t_{k}^{f}\right)$, where $\mu \left(t-t_{k}^{f}\right)$ is the step function starting at the firing time $t_{k}^{f}$, the solution for the inhibitory term is calculated as:

(16) $$\begin{array}{l} u_{j}^{in}\left(t\right)=\overset{m}{\underset{k\neq j}{\sum }}w_{kj}^{in}\underset{t_{k}^{f}}{\sum }e^{-\left(t-t_{k}^{f}\right)/\tau_{u}}\mu \left(t-t_{k}^{f}\right) \end{array}$$

where the first summation runs over all inhibitory synapses *k* and the second over all the firing times, $t_{k}^{f}$, of synapse *k*. The final solution for the membrane potential of neuron *j* is expressed as:

(17) $$\begin{array}{l} u_{j}\left(t\right)=\tau_{u}\beta \langle X,W_{j}^{ex}\rangle \left(1-e^{-t/\tau_{u}}\right)-\overset{m}{\underset{k\neq j}{\sum }}w_{kj}^{in}\underset{t_{k}^{f}}{\sum }e^{-\left(t-t_{k}^{f}\right)/\tau_{u}}\mu \left(t-t_{k}^{f}\right) \end{array}$$

Two terms control the change of the membrane potential in each neuron: a growing function (first term), that pulls the membrane potential up, and a regulatory function (the second term), that pulls the membrane potential down based on the previous (historic) spiking record of the recurrent signals (output spiking times). The spiking times are not given and are to be found progressively. Assuming that the times of all past recurrent spikes are known, $t_{j}^{f_{prev}}$, the time at which each neuron *j* will generate a spike is found by:

(18) $$t_{j}^{f_{next}}:u_{j}\left(t\right)=\vartheta_{j}|{\;}_{X,W_{j}^{ex},W_{j}^{in},t^{f_{prev}}}$$

Since the firing of a neuron affects the membrane potential of all other neurons, only the neuron that spiked first updates the membrane potentials of all other neurons. After that, the membrane potential of the neuron that fired is set to *u*_*r*_ and its recorded recurrent signals are emptied, $t_{j}^{f_{prev}}=0$.

#### 2.2.3. Learning Mechanisms

As shown in section 2.1.2, CSNN uses two types of learning mechanisms: adaptive firing threshold (Equation 5), and STDP rule (Equation 6). The adaptive firing threshold is used to establish the self-regulatory process that penalizes close firings of neurons. It depends directly on elapsed time (it decreases exponentially toward a resting value) and the neuron's firing events (it is increased by a constant value every time the neuron fires). In order to include the dynamics of the adaptive firing threshold into our model, we update Equation (18) by replacing ϑ_*j*_ for a time-dependent implementation $\vartheta_{j}\left(t\right)=\vartheta_{0}e^{-t/\tau_{\vartheta }}$:

(19) $$t_{j}^{f_{next}}:u_{j}\left(t\right)=\vartheta_{0}e^{-t/\tau_{\vartheta }}|{\;}_{X,W_{j}^{ex},W_{j}^{in},t^{f_{prev}}}$$

where ϑ_0_ is the initial firing threshold value, and τ_ϑ_ is the exponential decay constant. We also consider ϑ constant during calculation of the firing times (since τ_ϑ_ is normally much longer than the input presentation time) and update it only during firing events, which brings us back to Equation (18).

STDP enforces neuron affinity to similar input patterns. Every time a neuron fires, its synaptic connections are updated so the neuron will respond faster to similar future inputs. The synapse updates are based on the relative time difference between the receiving and the generated spikes. This mechanism was shown to be equivalent to the Expected Maximization (EM) algorithm (Nessler et al., 2009, 2013), which is an iterative procedure used for density model estimation of unlabeled data. At each iteration probability distribution functions are updated based on the current distribution of the given samples (sum of the samples weighted by its posterior probability). If normal distribution is used, the mean and variance update for unknown source *j* are calculated by:

(20) $$\begin{array}{l} \mu^{\prime }=\overset{n}{\underset{i=1}{\sum }}\frac{P\left(j|x^{\left(i\right)},\mu ,\Sigma \right)}{P\left(j\right)}x^{\left(i\right)} \end{array}$$

(21) $$\begin{array}{l} \Sigma_{j,k}^{\prime }=\overset{n}{\underset{i=1}{\sum }}\frac{P\left(j|x^{\left(i\right)},\mu ,\Sigma \right)}{P\left(j\right)}\left(x_{j}^{\left(i\right)}-\mu_{j}\right)\left(x_{k}^{\left(i\right)}-\mu_{k}\right) \end{array}$$

where *P*(*j*|*x*^(*i*)^) is the posterior probability of the source *j* given sample *x*^(*i*)^ and current distribution parameters μ and Σ. In the framework of CSNN, each spiking neuron (characterized by its weight vector *W*) represents one unknown source, and the parameter's update is based on the activity rate induced by the input *x* weighted by its probability of belonging to the spiking neuron. The EM algorithm can be simplified if the variance value is fixed to 1 and the posterior probability is discretized (0 or 1). Then, the update at each iteration is given by:

(22) $$\begin{array}{l} \mu_{j}^{\prime }=\frac{1}{r}\overset{r}{\underset{i=1}{\sum }}x_{j}^{\left(i\right)} \end{array}$$

where the summation operates only on the *r* number of samples that belong to neuron *j*. This is similar to the K-means algorithm. One more simplification is achieved by considering a sequential update process to accommodate for CSNN operation (sequential processing of samples):

(23) $$\begin{array}{l} \mu_{j}^{\prime }=\mu_{j}+\alpha x_{j}^{\left(i\right)} \end{array}$$

where α is an updating constant.

### 2.3. CRBA—A Competitive Rate-Based Algorithm

As we have seen, CSNN's operation can be modeled by a series of phases in which the change of the neuron's membrane potential, firing threshold, and connection weights are increased/decreased based on a neuron becoming, or not, a winner. Since the winner neuron, and the firing threshold and weight update at each phase are found by using Equations (18), (19), and (23), designing CRBA is straightforward and consists of these steps:

Step 1. Rank all neurons depending on their firing time. From Equations (17) and (18), we see that the ability of a neuron to fire depends on two terms: the excitatory term, controlled by the level of similarity between the neuron connection weights and the input sample, and the inhibitory term that is controlled by its record of all previous firings. Assuming that the inhibitory connections in CSNN are fixed and evenly distributed across all neurons, and that the firing threshold and weight updates can be neglected during a sample presentation (*t* < < τ_ϑ_ and weight update < < ϑ), it is possible to approximate in CRBA the competition process during each sample presentation using ranking of the neurons based on their spiking frequency calculated as the dot product between *X* and the neurons' weights *W* divided by the current firing threshold values.
Step 2. Select a winner neuron. Firing of neurons depends on the balance between the inhibitory and excitatory connections. If strong inhibition is used, only one neuron fires. At the time it fires, the inhibitory signal it sends to all other neurons strongly depresses their excitatory input which prevents them from firing for the remaining time of the sample presentation. On the other hand, when inhibition is weaker (soft inhibition), more than one neuron can fire (e.g., three fastest neurons) because the first spike event does not produce strong enough inhibition to completely shut down the other neurons.
Step 3. Calculate the number of spikes each winner neuron generates. This number can be calculated by using its frequency rate (from Step 1) scaled by a coefficient vector *C* of the form [*c*_1_, *c*_2_, ..., *c*_*n*_] where *c* is a coefficient based on the rank with *c*_1_ > *c*_2_ > ... > *c*_*n*_. For example, the coefficient vector *C* for three winner neurons may look like [1.0, 0.3, 0.1]. We select the coefficient values *c*_*i*_ for *n* number of winner neurons using $c_{i}=e^{-5i/n}$, which simulates a typical distribution of neuron firings observed in CSNN during a sample presentation.
Step 4. Update neuron's firing thresholds and weights. In this step the firing threshold and connection weights are updated for all winner neurons. This is done by first calculating the number of spikes the winner neurons may generate based on their ranking scores (Step 1). After that, the firing thresholds and weights of the winner neurons are updated using a constant increase (Equation 23), scaled by the predicted number of spikes. The non-winner neurons' firing thresholds are decreased based on an exponential decay function (specified before Equation 19).
Step 5. Weight normalization. The neurons' weights are normalized using Equation (7) (see section 2.1.2). This operation is done to limit the excitatory input each neuron receives. It also spreads the update through all its weights as the individual increases are averaged across all connections.

The pseudo-code for CRBA is shown as Algorithm 1.

**Algorithm 1:** Competitive Rate-based Algorithm (CRBA)

1: **Input**: *D, W*_0_, *m, n, t*, λ, α_*s*_, α_*w*_, α_ϑ_, ϑ_0_, ϑ_*r*_, τ_ϑ_, *C*
2: **Output**: *W*, ϑ
3: *W* ← *W*_0_, ϑ ← ϑ_0_ · ones(*m*)
4: **for** *X* in *D* **do**
5: $freq\leftarrow \frac{\langle X,W\rangle }{\vartheta }$
6: *winners* ← argsort(*freq*)[:*n*]
7: *spikes* ← α_*s*_ · *t* · *freq*[*winners*] · *C*
8: *W*[*winner*] ← *W*[*winner*] + α_*w*_ · *spikes* · *X*
9: $W\left[winner\right]\leftarrow \frac{\lambda }{W\left[winner\right].\text{sum()}}\cdot W\left[winner\right]$
10: ϑ[*winner*] ← ϑ[*winner*] + α_ϑ_ · *spikes*
11: $\vartheta \leftarrow \vartheta +\frac{\vartheta_{r}-\vartheta }{\tau_{\vartheta }}$
12: **end for**

The dataset, *D* = {*X*_1_, *X*_2_, ...*X*_*p*_}, initial weight matrix, *W*_0_, and eleven hyper-parameters: *m* (number of spiking neurons), *n* (number of winner neurons per sample presentation), *T* (sample running time), λ (total input weight), α_*s*/*w*/ϑ_ (scaling factors for number of spikes, weight update and firing threshold update), ϑ_0_ (initial firing threshold value), ϑ_*r*_ (firing threshold resting value), τ_ϑ_ (firing threshold time decay constant), and *C* (scaling vector) are the inputs to the algorithm.

In line 3 the neuron's weight and firing threshold values are initialized as the *p* × *m* dimensional matrix *W* and *m*-dimensional vector ϑ, respectively. The FOR loop presents each input sample *X*. First, winner neurons are found based on the spiking frequency rate (lines 5 and 6). Then the frequency is scaled by vector *C* and the coefficient α_*s*_ to compute the number of spikes each winner neuron generates during the presentation time *t* (line 7). After that, the estimated number of spikes is used to update the weight of the winner neuron (line 8 and 9) and its firing threshold value (line 10). Line 11 implements the firing threshold's exponential decay.

#### 2.3.1. Parameter Selection

We use CSNN operation as guidance for choosing CRBA's hyper-parameters. At initialization, CRBA internal parameters (*t*, *u*_*r*_, λ, ϑ_0_, and ϑ_*r*_ and τ_ϑ_) are set with values that are the same as the ones used in CSNN, and the scaling factors (α_*s*_, α_*w*_, and α_ϑ_) are chosen in such a way that the predicted number of spikes and the weight and firing threshold updates for the winner neurons match the ones in CSNN (around 30 spikes for the winner neuron, and 0.3 and 2 mV for weight and firing threshold increases, respectively, per sample presentation).

Regarding selection of the number of winner neurons, it has been shown that the stronger the inhibition in CSNN the better it performs (1–3 winner neurons during each sample presentation) (Diehl and Cook, 2015). Our experiments have shown that CRBA mimics this characteristic and that it performs best when only one winner neuron is selected (see section 3.5).

To initialize the weights, *W*, we use sample-based initialization that was shown to reduce the number of samples needed for learning (Cachi et al., 2020).

#### 2.3.2. Operation

CRBA approximation of CSNN operation is based on the interplay of three mechanisms: neuron competition, variable firing threshold, and weights update. The difference is that CRBA uses only one loop iteration (five steps) per sample instead of the dynamic operation used in CSNN. The details of how these mechanisms interplay are as follows. First, at initialization, every neuron is assigned a weight vector *W* and a firing threshold value ϑ to be used during sample presentations. The closer the sample input vector, *X*, is to a neuron's weight vector, *W*, the stronger is its calculated spiking frequency (line 5) and it has higher chance to be selected as a winner neuron (line 6). Note that at initialization the division operation to calculate the spiking frequency (line 6) is irrelevant since all neurons start with the same firing threshold value, ϑ (from section 2.3.1). After ranking, only the winner neurons' weights and firing thresholds are updated. The weight update (line 8) modifies the *W* so it moves closer to the presented input sample *X*. This makes the neuron more selective to the class *X* belongs to (its spiking frequency for a sample of the same class will be also high), since it is assumed that samples from the same class come from the same distribution, in the *P* dimensional space. The firing threshold update has the opposite effect (line 10). Higher firing thresholds reduce the calculated spiking frequency. This process counteracts the weight update and has the effect of controlling that the same neurons are not continuously becoming winners. This controlling effect is further strengthened by using the number of spikes to scale the firing threshold update (line 10) and by reducing the non-winning neurons' firing thresholds (line 11). The interplay between weights and firing thresholds defines a convergence behavior of the system (time at which the positive effect of the weight update and negative effect of the firing threshold update cancel each other). After a number of samples are presented, it is expected that the mean firing threshold of all neurons increases and the calculated number of spikes decreases. Regarding the neuron's weights, they learn class patterns as they move toward the centroid of a cluster of the samples in the *P* dimensional space.

#### 2.3.3. Usage

CRBA, similarly to CSNN, can be used for unsupervised classification. It is done in two phases: learning and testing. The learning phase uses input data to, first, tune the competitive neurons in a completely unsupervised way (i.e., find weights and firing thresholds using Algorithm 1) and, second, assign labels to the already tuned neurons based on the per-class neuron activity (using labeled samples). In the testing phase, the already labeled neurons are used to predict the labels for new unseen samples using a maximum-voting scheme (Querlioz et al., 2011; Diehl and Cook, 2015; Hazan et al., 2018; Cachi et al., 2020), or using an add-on classifier, such as a fully connected feedfoward neural network (Demin and Nekhaev, 2018; Kheradpisheh et al., 2018; Krotov and Hopfield, 2019).

After CRBA final parameters (the weights and firing thresholds) are found they can be used to initialize the corresponding parameters of CSNN. The weights are transferred to CSNN without any modification because both networks use the same normalization operation. A re-scaling operation, however, is required for transferring the firing threshold since CRBA generates higher threshold values than CSNN (see section 3.1.2). Additionally, it was found that the stability of CSNN+CRBA depends on the values of the firing thresholds. Small firing threshold values allow for generation of more spikes during sample presentations, which results in considerable changes of the weights and lower accuracy. High firing threshold values have the opposite effect: they reduce the number of spikes and small changes of the weights, which makes the network stable. Specifically, the thresholds found by CRBA were re-scaled to match the thresholds of CSNN trained with 200 K samples (i.e., threshold were re-scaled to the range of 35–60 mV).

## 3. Results

The performance of CRBA is tested on the MNIST (LeCun and Cortes, 2010) and Fashion-MNIST (Xiao et al., 2017) datasets (each dataset contains 60 K training and 10 K testing samples of size 28 × 28 pixels). The experiments are divided into five parts. First, we analyze learning performance of CRBA (in terms of accuracy, and firing threshold and weight evolution) and compare it with performance of CSNN's implementation that uses sample-based initialization of weights (Cachi et al., 2020) (details are provided in the Supplementary Material). Second, we analyze usage of weights and firing thresholds found by CRBA to initialize CSNN (CSNN+CRBA). Third, we compare testing accuracy of CRBA, CSNN, and CSNN+CRBA. Fourth, we compare the algorithms' running times and, lastly, CRBA is compared with unsupervised spiking neural networks. To select the hyper-parameters the process described in section 2.3.1 is used and they are listed in Table 1.

**Table 1:** CRBA's hyper-parameters.

*m*	100, 400, 1,600
*n*	1
*T*	350
λ	1
α_*s*_	10
α_*w*_	0.00005
α_ϑ_	0.05
ϑ_0_	20
ϑ_*r*_	−10
τ_ϑ_	10^5, 10^6, 10^7

All the presented results are the average of 10 runs using 100, 400, and 1,600 neurons, with maximum-voting inference method (Diehl and Cook, 2015), for both CRBA and CSNN (on an Intel Core i9-9900K machine with 64 GB RAM). All the code is posted at GitHub^1^.

### 3.1. CRBA

#### 3.1.1. Accuracy on Validation Data During Learning

**Figure 3:**
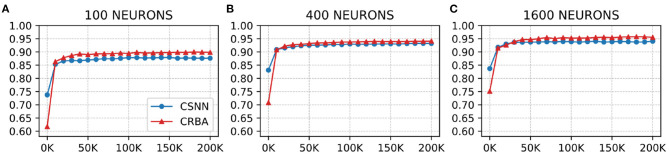
CRBA and CSNN accuracy on MNIST data vs. number of samples using **(A)** 100 neurons, **(B)** 400 neurons, and **(C)** 1600 neurons.

**Figure 4:**
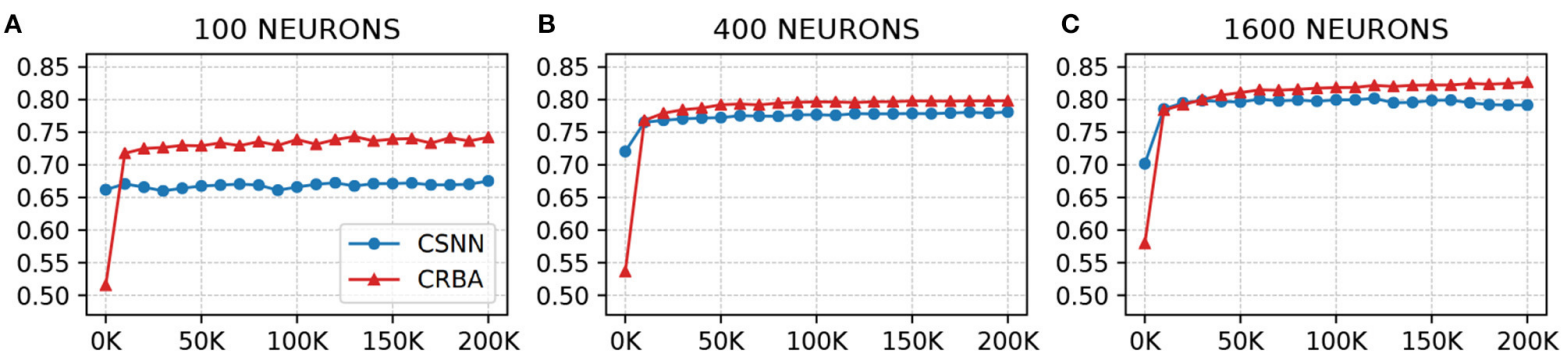
CRBA and CSNN accuracy on Fashion-MNIST data vs. number of samples using **(A)** 100 neurons, **(B)** 400 neurons, and **(C)** 1600 neurons.

We use a 10 K validation set, drawn from the learning dataset, to show the evolution of learning accuracy after 10 K sample presentations, in the range from 0 to 200 K (i.e., 10 K, second 10 K, third 10 K, etc.). Figure 3 plots validation results for CRBA and CSNN using (A) 100 neurons, (B) 400 neurons, and (C) 1,600 neurons on MNIST data. Figure 4 shows the same experiments for Fashion-MNIST data.

Figures 3, 4 show that CRBA performs on par or slightly better than CSNN, for all configurations, on both datasets. At the beginning, it starts with lower accuracy than CSNN, however, the difference quickly decreases and at after around 15 K sample presentations it achieves a higher accuracy. After 50 K samples both curves reach a plateau. Note that both algorithms achieve better accuracy as more neurons are used, however the gain when using 1,600 neurons instead of 400 neurons is smaller than the ones observed when switching from 100 to 400 neurons.

#### 3.1.2. Network Dynamics

**Figure 5:**
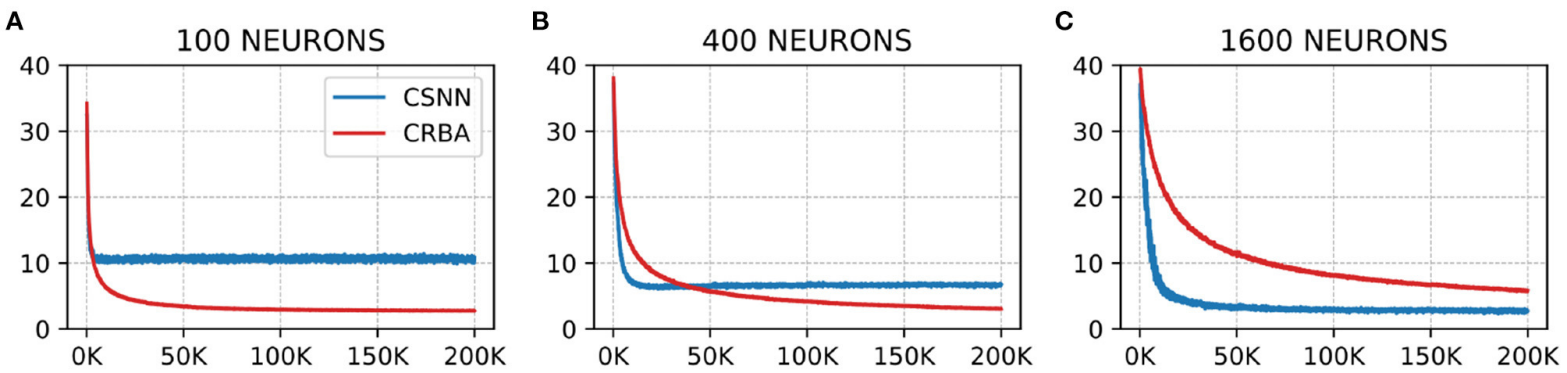
Number of spikes vs. number of samples on MNIST data using **(A)** 100 neurons, **(B)** 400 neurons, and **(C)** 1600 neurons.

**Figure 6:**
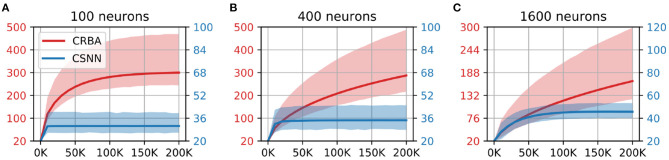
Firing threshold (mV) vs. number of samples on MNIST data using **(A)** 100 neurons, **(B)** 400 neurons, and **(C)** 1600 neurons.

We compare the evolution of CRBA's number of spikes of the winner neuron, firing threshold and connection weights during learning with that of CSNN's. Figures 5, 6 show the number of spikes and firing threshold, respectively, for CRBA and CSNN using 100 (A), 400 (B), and 1,600 (C) neurons on MNIST data. Note that Figure 6 plots the average firing threshold for all neurons (bold line) and the area surrounding the minimum and maximum firing threshold is shown in colors; it also uses two different y-ax scales: on the left-hand side for CRBA and on the right-hand side for CSNN.

We see that the evolution of the number of spikes and firing threshold for both CSNN and CRBA follow a similar pattern: exponential decrease in the number of spikes and increase of the firing threshold. Specifically, we observe that the number of spikes after presentation of 200 K samples in CRBA is about 2.5, 3, and 6 for 100, 400, and 1,600 spiking neurons while in CSNN it is 11, 7, and 2. Regarding the firing threshold, the average for CRBA increases to around 300 mV for 100 and 400 neurons used, and to 150 mV for 1,600 neurons while CSNN's firing threshold stays below 50 mV. The explanation for CRBA reaching higher firing threshold is that CRBA uses a simple linear equation to determine the number of spikes, while CSNN uses an exponential membrane potential function.

**Figure 7:**
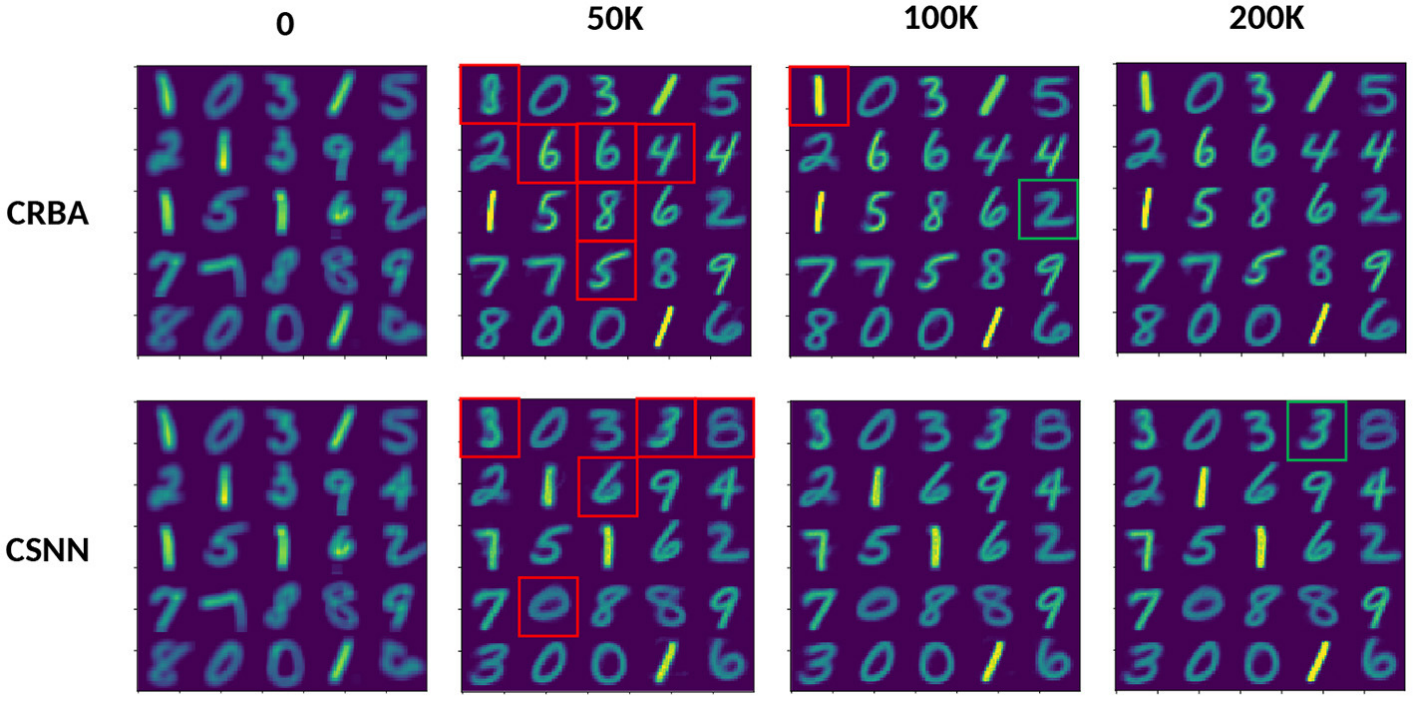
CRBA and CSNN weights comparison after presenting 0 through 200 K input samples; each window is constructed by displaying the weight vectors.

Figure 7 compares the weight vector, *W*, of 25 neurons (the first 25 out of 400) initialized with sample values at 0, 50, 100, and 200 K sample presentations on MNIST data, for both CRBA and CSNN. To visualize the weights in a visual way, all 25 *W* vectors (one for each neuron) are displayed as windows of 28 × 28 pixels, each corresponding to part of the image where a digit is displayed (e.g., in red boxes).

We see that the evolution of weights is similar for CRBA and CSNN. After 50 K sample presentations, weights in CRBA and CSNN show a considerable difference from their initial weights (e.g., see the red boxed weights that most changed). This happens because at initialization the neurons are just beginning to learn to represent different classes. There are no major changes in the weights from 50 to 200 K (only the first neuron in CRBA changed from an 8 to a 1). However, with more sample presentations we see that the values are more pronounced; see the green framed weight that changed from a blurred “2” to a better defined one for CRBA, and “3” for CSNN.

### 3.2. CSNN+CRBA

**Figure 8:**
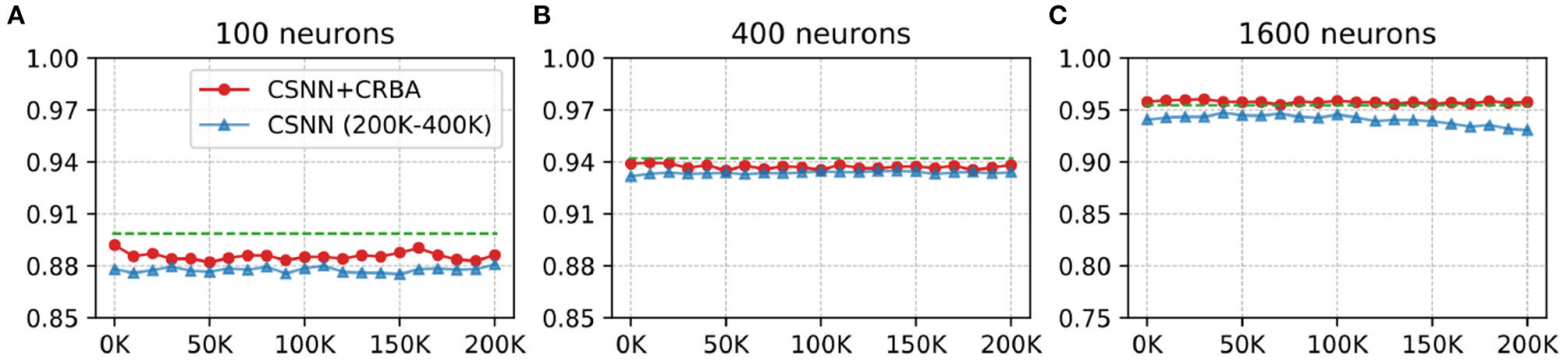
CSNN+CRBA, CSNN and CRBA (dashed lines) accuracies vs. number of input samples on MNIST dataset using **(A)** 100 neurons, **(B)** 400 neurons, and **(C)** 1600 neurons.

**Figure 9:**
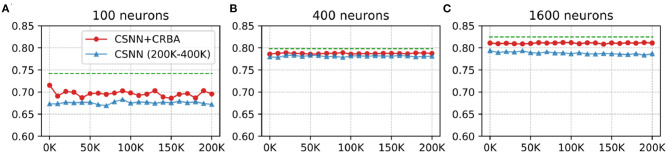
CSNN+CRBA, CSNN and CRBA (dashed lines) accuracies vs. number of input samples on Fashion-MNIST dataset using **(A)** 100 neurons, **(B)** 400 neurons, and **(C)** 1600 neurons.

Here we test performance of using parameters found by CRBA (weights and firing thresholds) when they are used to initialize CSNN parameters; we call this configuration CSNN+CRBA. Figures 8, 9 show accuracies during 4 epochs of training for CSNN+CRBA (we used parameters found by CRBA using 200 K learning samples), for 100 (A), 400 (B), and 1,600 (C) neurons at different moments of training on MNIST and Fashion-MNIST datasets. CSNN results of training with 200 to 400 K samples and CRBA accuracy after using 200K training samples (dashed lines) are also shown for comparison.

We see that CSNN+CRBA immediately starts with high accuracy and remains stable (does not increase nor decrease) with further training. CSNN+CRBA shows a slight reduction of accuracy, when compared with CRBA, but it is still higher than normally trained CSNN. These two observations show that by initializing CSNN with parameters learned by CRBA we avoid very long learning time of CSNN and still achieve better accuracy. This approach will become very useful in the near future when CSNN can be implemented on neuromorphic processors that do not have the ability to perform on-chip learning.

### 3.3. Accuracy on Test Data

Tables 2, 3 compare accuracy of CSNN, CRBA, and CSNN+CRBA for MNIST and Fashion-MNIST test datasets, after training with 0, 2, 5, 10, 20, 50, 100, and 200 K samples. CSNN+CRBA was initialized using parameters found by CRBA using the just mentioned number of samples (no additional training of CSNN+CRBA). We did it because additional training does not significantly improve accuracy (see section 3.2).

**Table 2:** Testing accuracy on MNIST data.

		**Number of samples**							
**Neurons**	**Method**	**0**	**2 K**	**5 K**	**10 K**	**20 K**	**50 K**	**100 K**	**200 K**
100	CSNN	**74.52**	**82.97**	**84.96**	85.17	85.38	86.17	86.93	86.19
	CRBA	60.92	82.01	83.83	**85.83**	**87.31**	**88.49**	**88.96**	**89.29**
	CSNN+CRBA	72.92	83.10	84.23	85.30	86.27	86.96	87.20	87.49
400	CSNN	**82.44**	**88.37**	**89.42**	**90.42**	90.97	91.87	91.99	92.11
	CRBA	70.35	84.92	89.06	89.76	**91.65**	**92.77**	**93.35**	**93.95**
	CSNN+CRBA	79.87	87.02	88.81	89.90	90.93	91.86	92.45	92.91
1,600	CSNN	**83.19**	86.23	88.89	91.36	92.75	93.38	93.50	93.84
	CRBA	74.22	**87.39**	**89.90**	**91.47**	**93.02**	**94.41**	**95.04**	95.44
	CSNN+CRBA	78.30	86.76	89.42	90.86	92.76	94.00	95.01	**95.48**

*Bold values denote the highest accuracies achieved at the specified number of training samples for each configuration (100, 400 and 1600 neurons)*.

**Table 3:** Testing accuracy on Fashion-MNIST data.

		**Number of samples**							
**Neurons**	**Method**	**0**	**2 K**	**5 K**	**10 K**	**20 K**	**50 K**	**100 K**	**200 K**
100	CSNN	**65.16**	67.66	65.37	65.89	64.47	65.59	65.54	65.63
	CRBA	51.58	69.82	**70.69**	**71.39**	**71.98**	**72.42**	**73.45**	**73.95**
	CSNN+CRBA	52.51	**70.02**	70.44	71.27	71.57	71.96	71.91	71.54
400	CSNN	**71.04**	**74.95**	**75.35**	75.61	75.68	76.05	76.66	77.25
	CRBA	53.56	72.71	75.02	**76.22**	**77.67**	**78.90**	**79.23**	**79.54**
	CSNN+CRBA	51.62	70.63	74.73	75.92	77.62	78.71	79.03	79.11
1,600	CSNN	**69.35**	73.14	75.04	77.10	78.49	78.96	78.58	78.56
	CRBA	57.63	**73.62**	**76.97**	**77.78**	**78.93**	**80.71**	**81.43**	**82.13**
	CSNN+CRBA	36.82	54.65	61.26	65.41	70.39	76.79	80.56	81.49

*Bold values denote the highest accuracies achieved at the specified number of training samples for each configuration (100, 400 and 1600 neurons)*.

We can draw two conclusions from these results. First, similar to validation accuracy shown above, CRBA performs better (1–8%) than CSNN when trained with 20K or more samples. Second, CSNN+CRBA has slightly lower accuracy (0.5–2%) than CRBA, but higher (0.5–6%) than pure CSNN, in all configurations. The former phenomenon has been also observed by others, for instance when deep learning algorithms are used to find weights for networks of spiking neurons (Diehl et al., 2015; Rueckauer et al., 2017).

### 3.4. Running Time

CRBA was designed with the goal of significantly improving the run time of CSNN. Table 4 shows comparison of running times of CRBA with CSNN implementations using 100, 400, and 1,600 neurons at one epoch of learning (using 50 K samples) on MNIST dataset.

**Table 4:** Running time comparisons.

		**Time (sec)**		
**Neurons**	**Method**	**Learning**		**Testing**
		**Parameter tuning**	**Neuron labeling**	
100	CSNN	14,785.9	9,865.4	1,647.9
	CRBA	**3.9**	**0.3**	**0.1**
	CSNN+CRBA	3.9	0.3	1,628.0
400	CSNN	18,134.5	10,150.6	1,725.5
	CRBA	**11.8**	**0.9**	**0.2**
	CSNN+CRBA	11.8	0.9	1,745.1
1,600	CSNN	161,148.9	13,710.7	2,124.7
	CRBA	**56.4**	**5.1**	**0.9**
	CSNN+CRBA	56.4	5.1	2,140.3

*Bold values denote the fastest running times for the parameter tunning, neuron labeling, or testing phase for each network configuration (100, 400 and 1600 neurons)*.

Note that CRBA requires only about 4, 13, and 60 s while CSNN implementations require more than 7, 8, and 49 h for the three different numbers of neurons used. This result was expected since CRBA processes each input in just one iteration, while CSNN runs each sample using 3,500 time steps (350 ms with 0.1 ms time step).

Using CRBA-found weights in CSNN avoids entirely its learning phase, which means that CSNN can be used directly for making predictions. Recall, as described above and shown in Table 4, that both CRBA and CSNN operations consist of two phases: learning (tuning the network parameters and neuron labeling) and testing. Obviously, the testing time in CSNN cannot be avoided but major saving comes from no need of tuning its parameters during learning, especially when larger size of the network is used, such as 1,600 neurons. CSNN+CRBA testing still requires orders of magnitude more time than CRBA.

### 3.5. CRBA With Different Number of Winner Neurons

A general method for constructing multi-layer CSNN architectures is yet to be developed. However, since the proposed CRBA is a very fast approximation of CSNN that can be run on regular computers, one can ask a question if it can be used in a multi-layer architecture. Note that the output of the competitive spiking layer is very sparse (only few neurons fire) which basically prohibits stacking additional layers (e.g., in a CSNN with 400 spiking neurons, only a couple of neurons fire during each sample presentation). One way to address this sparsity is to decrease inhibition to allow more neurons to fire, which in CRBA means increasing the number of winner neurons per sample presentation (all presented so far results used only one winner neuron). Table 5 shows testing accuracy of CRBA on MNIST data while experimenting with different number of winner neurons.

**Table 5:** CRBA accuracy with different number of winner neurons.

	**Number of winner neurons**					
**Neurons**	**1**	**% of the total**				
		**1%**	**2.5%**	**5%**	**10%**	**25%**
100	89.29	89.29	**89.43**	87.04	80.92	65.13
400	**93.95**	93.75	92.58	90.00	81.16	66.58
1,600	**95.44**	95.06	93.42	89.97	81.55	66.71

*Bold values denote the highest accuracies achieved for each network configuration (100, 400 and 1600)*.

It shows that in general the accuracy decreases as the number of winner neurons is increased. This effect might be due to the fact that, as discussed in section 2.3.2, the update at each sample presentation moves the weight vector of the winner neurons closer to the input sample vector. Thus, if more than one neuron is updated, all of them move toward the same input pattern which impacts their ability to learn variations of patterns within the same class (like different ways of writing digit “2”). This means that reducing the inhibition is not a sufficient mechanism for constructing multi-layer architectures.

### 3.6. Comparison With Other Spiking Neural Networks

Since CRBA is rate-based approximation of CSNN it can be compared directly only with other unsupervised spiking neural networks, which use STDP learning rule or a variation of it. As explained in section 2.3.3, these networks require a learning phase that consist of (a) unsupervised learning of weights, and (b) labeling of the spiking neurons. It is followed by a testing phase that uses some add-on inference method to predict labels for new data samples. Table 6 shows comparison of CRBA with some current unsupervised spiking neural networks. It also shows results for spiking neural networks which are trained with a supervised, reward-modulated STDP rule. The results are shown for two CRBA configurations and for CSNN+CRBA. One CRBA configuration uses 1,600 neurons and maximum-voting inference method. The other uses 2,000 neurons using an add-on fully supervised neural network with two hidden layers (of 2,000 and 200 neurons), 0.2 dropout for CRBA, no hidden layers for CSNN+CRBA, softmax classifier, and Adam Optimizer as the inference method.

**Table 6:** Comparison of accuracy on MNIST dataset.

**Architecture/Learning rule**	**Operation**	**Learning**	**Inference**	**Neurons**	**Accuracy (%)**
CSNN/discrete STDP (Querlioz et al., 2013)	Spike-based	Unsupervised	Maximum voting	300	93.5
CSNN/triplet STDP (Diehl and Cook, 2015)	Spike-based	Unsupervised	Maximum voting	6,400	95.0
Self organizing CSNN / STDP (Hazan et al., 2018)	Spike-based	Unsupervised	N-gram voting	1,600	94.1
Six layer conv-CSNN + SVM / STDP (Kheradpisheh et al., 2018)	Spike-based	Unsupervised	Add-on classifier	–	98.4
CSNN/maximization neural activity (Demin and Nekhaev, 2018)	Rate-based	Supervised	–	400	96.2
CSNN + Fully Con. NN / EM (Krotov and Hopfield, 2019)	Rate-based	Unsupervised	Add-on classifier	2,000	98.6
Six layer conv-CSNN + / reward modulated STDP (Mozafari et al., 2019)	Spike-based	Supervised	–	–	97.2
Two layer SNN / alpha synaptic function (Comsa et al., 2020)	Spike-based	Supervised	–	340	98.0
CSNN (Cachi et al., 2020)	Spike-based	Unsupervised	Max-voting	1,600	93.8
CRBA	Rate-based	Unsupervised	Max-voting	1,600	95.4
CRBA + Fully Con. NN	Rate-based	Unsupervised	Add-on classifier	2,000	98.0
CSNN + CRBA	Rate-based	Unsupervised	Max-voting	1,600	95.5
CSNN + CRBA + Fully Con. NN	Rate-based	Unsupervised	Add-on classifier	2,000	96.3

We notice that CRBA performs better than networks using voting inference, and it is third best among networks that use add-on classifiers. Note that the network that achieves the highest accuracy (Krotov and Hopfield, 2019) is also a rate-based algorithm similar to ours but that uses a general form of EM algorithm while we use a discrete version of it. The general EM algorithm used in Krotov and Hopfield (2019) produces negative weights that are impossible to use in CSNN architecture. We chose the discrete implementation of EM algorithm because our aim was to develop a rate-based approximation of CSNN. As shown by the experiments, CRBA is fully compatible with CSNN, meaning that CRBA-learned weights and firing thresholds can be directly used to initialize CSNN: CSNN+CRBA.

The second-best network (Kheradpisheh et al., 2018) is a spiking architecture consisting of six-layer convolutional CSNN (3 feed-forward convolutional-competitive layers plus 3 max pooling layers). Although CRBA cannot be used to approximate the multilayer architecture used in Kheradpisheh et al. (2018), it can serve as a building block for future investigations.

Regarding CSNN+CRBA, we notice that in spite transfer of weights and firing thresholds, it performs better than the other normally trained spiking networks shown in Table 6. Importantly, in CSNN+CRBA, using CRBA learned weights and firing thresholds we skip performing CSNN's learning phase that requires very long computational time.

## 4. Discussion

In this paper we introduced a new algorithm, CRBA, that implements the rate-based operation of CSNN. CRBA uses similarity between the input and synapse vectors to predict the network's firing output, without the need of running temporal simulations carried out by spiking neural networks. It also uses discrete implementation of the EM algorithm as the learning mechanism. The result of doing it was a significant reduction of computational time by more than three orders of magnitude, and a slight improvement of accuracy while also maintaining its compatibility with CSNN.

Using CRBA on the MNIST and Fashion-MNIST datasets reduced the total learning and testing time from around 7, 8, and 49 h to mere seconds: 4.3, 12.9, and 62.4 s, when using 100, 400, and 1,600 neurons, respectively. At the same time it had slightly higher accuracies on both MNIST (1–3%) and Fashion-MNIST (2–8%) datasets.

It is important to stress that CRBA is fully compatible with CSNN, meaning that the synaptic weights and firing thresholds learned by CRBA can be used, without any changes, in CSNN. This is a major advantage of CRBA over other models. Experimentally, we found that transferring CRBA parameters to CSNN resulted in accuracy slightly lower than CBRA's but better than those obtained with normal CSNN learning. The major benefit of avoiding CSNN learning is that it can be directly used for testing (without further learning). This result will play significant role in a near future when CSNN can be deployed on the low-energy neuromorphic computers.

CRBA may inspire a wider use of competitive spiking neural networks since it greatly simplifies their operation while maintaining accuracy. Importantly, CRBA retains key advantage of competitive spiking neural networks, namely, it performs unsupervised selection of the most important input features for classification tasks on real-valued data. The ideas presented in this paper provide background for CRBA's extensions like its possible use in the framework of spiking convolutional neural networks (Tavanaei and Maida, 2017).

## Data Availability Statement

The original contributions generated for the study are included in the article/Supplementary Material, further inquiries can be directed to the corresponding author/s.

## Author Contributions

PC, SV, and KC contributed to conception and design of the study. PC organized the database, performed the statistical analysis, and wrote the first draft of the manuscript. PC and KC wrote sections of the manuscript. All authors contributed to manuscript revision, read, and approved the submitted version.

## Conflict of Interest

The authors declare that the research was conducted in the absence of any commercial or financial relationships that could be construed as a potential conflict of interest.

## Footnotes

^1^https://github.com/PaoloGCD/Rate-based-CSNN

## References

[B1] Brette R. Gerstner W. (2005). Adaptive exponential integrate-and-fire model as an effective description of neuronal activity. J. Neurophysiol. 94, 3637–3642. 10.1152/jn.00686.2005 16014787

[B2] Cachi P. G. Ventura S. Cios K. J. (2020). “Fast convergence of competitive spiking neural networks with sample-based weight initialization,” in Information Processing and Management of Uncertainty in Knowledge-Based Systems, eds M.-J. Lesot, S. Vieira, M. Z. Reformat, J. P. Carvalho, A. Wilbik, B. Bouchon-Meunier, and R. R. Yager (Cham: Springer International Publishing), 773–786. 10.1007/978-3-030-50153-2_57

[B3] Ciregan D. Meier U. Schmidhuber J. (2012). “Multi-column deep neural networks for image classification,” in 2012 IEEE Conference on Computer Vision and Pattern Recognition (Providence, RI), 3642–3649. 10.1109/CVPR.2012.6248110

[B4] Comsa I. M. Potempa K. Versari L. Fischbacher T. Gesmundo A. Alakuijala J. (2020). “Temporal coding in spiking neural networks with alpha synaptic function,” in ICASSP 2020 - *2020 IEEE International Conference on Acoustics, Speech and Signal Processing (ICASSP)* (Barcelona), 8529–8533. 10.1109/ICASSP40776.2020.9053856

[B5] Demin V. Nekhaev D. (2018). Recurrent spiking neural network learning based on a competitive maximization of neuronal activity. Front. Neuroinform. 12:79. 10.3389/fninf.2018.00079 30498439 PMC6250118

[B6] Diehl P. Cook M. (2015). Unsupervised learning of digit recognition using spike-timing-dependent plasticity. Front. Comput. Neurosci. 9:99. 10.3389/fncom.2015.00099 26941637 PMC4522567

[B7] Diehl P. U. Neil D. Binas J. Cook M. Liu S. Pfeiffer M. (2015). “Fast-classifying, high-accuracy spiking deep networks through weight and threshold balancing,” in 2015 International Joint Conference on Neural Networks (IJCNN) (Killarney), 1–8. 10.1109/IJCNN.2015.7280696

[B8] Ermentrout B. (1998). Linearization of F-I curves by adaptation. Neural Comput. 10, 1721–1729. 10.1162/089976698300017106 9744894

[B9] Gerstner W. Kistler W. M. (2002). Mathematical formulations of Hebbian learning. Biol. Cybern. 87, 404–415. 10.1007/s00422-002-0353-y 12461630

[B10] Gerstner W. Kistler W. M. Naud R. Paninski L. (2014). Neuronal Dynamics: From Single Neurons to Networks and Models of Cognition. New York, NY: Cambridge University Press. 10.1017/CBO9781107447615

[B11] Hazan H. Saunders D. Sanghavi D. T. Siegelmann H. Kozma R. (2018). “Unsupervised learning with self-organizing spiking neural networks,” in 2018 International Joint Conference on Neural Networks (IJCNN) (Rio de Janeiro). 1–6. 10.1109/IJCNN.2018.8489673

[B12] Hebb D. O. (1949). The Organization of Behavior: A Neuropsychological Theory. New York, NY: John Wiley; Chapman & Hall.

[B13] Kandel E. R. Schwartz J. H. Jessell T. M. Jessell M. B. T. Siegelbaum S. Hudspeth A. (2013). Principles of Neural Science, Vol. 5. New York, NY: McGraw-Hill.

[B14] Kheradpisheh S. R. Ganjtabesh M. Thorpe S. J. Masquelier T. (2018). Stdp-based spiking deep convolutional neural networks for object recognition. Neural Netw. 99, 56–67. 10.1016/j.neunet.2017.12.005 29328958

[B15] Koch C. Segev I. (1998). Methods in Neuronal Modeling: From Ions to Networks. Cambridge, MA: MIT Press.

[B16] Konorski J. (1948). Conditioned Reflexes and Neuron Organization. Cambridge: Cambridge University Press.

[B17] Krotov D. Hopfield J. J. (2019). Unsupervised learning by competing hidden units. Proc. Natl. Acad. Sci. U.S.A. 116, 7723–7731. 10.1073/pnas.1820458116 30926658 PMC6475390

[B18] LeCun Y. Cortes C. (2010). MNIST handwritten digit database. Available online at: http://yann.lecun.com/exdb/mnist/

[B19] Liang Z. Schwartz D. Ditzler G. Koyluoglu O. O. (2018). The impact of encoding-decoding schemes and weight normalization in spiking neural networks. Neural Netw. 108, 365–378. 10.1016/j.neunet.2018.08.024 30261415

[B20] Lobov S. A. Chernyshov A. V. Krilova N. P. Shamshin M. O. Kazantsev V. B. (2020). Competitive learning in a spiking neural network: towards an intelligent pattern classifier. Sensors 20:500. 10.3390/s20020500 31963143 PMC7014236

[B21] Masquelier T. Thorpe S. J. (2007). Unsupervised learning of visual features through spike timing dependent plasticity. PLoS Comput. Biol. 3:e30031. 10.1371/journal.pcbi.0030031 17305422 PMC1797822

[B22] Morrison A. Diesmann M. Gerstner W. (2008). Phenomenological models of synaptic plasticity based on spike timing. Biol. Cybern. 98, 459–478. 10.1007/s00422-008-0233-1 18491160 PMC2799003

[B23] Mozafari M. Ganjtabesh M. Nowzari-Dalini A. Thorpe S. J. Masquelier T. (2019). Bio-inspired digit recognition using reward-modulated spike-timing-dependent plasticity in deep convolutional networks. Pattern Recogn. 94, 87–95. 10.1016/j.patcog.2019.05.015

[B24] Nessler B. Pfeiffer M. Buesing L. Maass W. (2013). Bayesian computation emerges in generic cortical microcircuits through spike-timing-dependent plasticity. PLoS Comput. Biol. 9:e1003037. 10.1371/journal.pcbi.1003037 23633941 PMC3636028

[B25] Nessler B. Pfeiffer M. Maass W. (2009). “STDP enables spiking neurons to detect hidden causes of their inputs,” in Advances in Neural Information Processing Systems 22, eds Y. Bengio, D. Schuurmans, J. D. Lafferty, C. K. I. Williams, and A. Culotta (Red Hook: Curran Associates, Inc.), 1357–1365.

[B26] Pfister J.-P. Gerstner W. (2006). Triplets of spikes in a model of spike timing-dependent plasticity. J. Neurosci. 26, 9673–9682. 10.1523/JNEUROSCI.1425-06.2006 16988038 PMC6674434

[B27] Querlioz D. Bichler O. Dollfus P. Gamrat C. (2013). Immunity to device variations in a spiking neural network with memristive nanodevices. IEEE Trans. Nanotechnol. 12, 288–295. 10.1109/TNANO.2013.2250995

[B28] Querlioz D. Bichler O. Gamrat C. (2011). “Simulation of a memristor-based spiking neural network immune to device variations,” in The 2011 International Joint Conference on Neural Networks (San Jose, CA), 1775–1781. 10.1109/IJCNN.2011.6033439

[B29] Rueckauer B. Lungu I.-A. Hu Y. Pfeiffer M. Liu S.-C. (2017). Conversion of continuous-valued deep networks to efficient event-driven networks for image classification. Front. Neurosci. 11:682. 10.3389/fnins.2017.00682 29375284 PMC5770641

[B30] Rumelhart D. E. Zipser D. (1985). Feature discovery by competitive learning. Cogn. Sci. 9, 75–112. 10.1207/s15516709cog0901_5

[B31] Shin J. Smith D. Swiercz W. Staley K. Rickard J. T. Montero J.. (2010). Recognition of partially occluded and rotated images with a network of spiking neurons. IEEE Trans. Neural Netw. 21, 1697–1709. 10.1109/TNN.2010.2050600 21047704

[B32] Tavanaei A. Maida A. S. (2017). “Multi-layer unsupervised learning in a spiking convolutional neural network,” in 2017 International Joint Conference on Neural Networks (IJCNN) (Anchorage, AK), 2023–2030. 10.1109/IJCNN.2017.7966099

[B33] Wan L. Zeiler M. Zhang S. Cun Y. L. Fergus R. (2013). “Regularization of neural networks using dropconnect,” in Proceedings of the 30th International Conference on Machine Learning, Volume 28 of Proceedings of Machine Learning Research, eds S. Dasgupta and D. McAllester (Atlanta, GA: PMLR), 1058–1066.

[B34] Wysoski S. G. Benuskova L. Kasabov N. (2008). Fast and adaptive network of spiking neurons for multi-view visual pattern recognition. Neurocomputing 71, 2563–2575. 10.1016/j.neucom.2007.12.038

[B35] Xiao H. Rasul K. Vollgraf R. (2017). Fashion-MNIST: a novel image dataset for benchmarking machine learning algorithms. arXiv [Preprint] arXiv:1708.07747.

[B36] Zenke F. Agnes E. J. Gerstner W. (2015). Diverse synaptic plasticity mechanisms orchestrated to form and retrieve memories in spiking neural networks. Nat. Commun. 6:6922. 10.1038/ncomms7922 25897632 PMC4411307

[B37] Zylberberg J. Murphy J. T. DeWeese M. R. (2011). A sparse coding model with synaptically local plasticity and spiking neurons can account for the diverse shapes of v1 simple cell receptive fields. PLoS Comput. Biol. 7:e1002250. 10.1371/journal.pcbi.1002250 22046123 PMC3203062

