# Supplementary material for: CRBA: A Competitive Rate-Based Algorithm Based on Competitive Spiking Neural Networks
Source: Front Comput Neurosci. 2021 Apr 22;15:627567. doi: 10.3389/fncom.2021.627567 (PMC8100331; doi:10.3389/fncom.2021.627567)
Supplement: Supplementary file 1 [file Data_Sheet_1.PDF]

CSNN's equations and hyper-parameters

Membrane potential

$$\tau_u \frac{du}{dt} = u_0 - u + g_e(u_{ge} - u) + g_i(u_{gi} - u)$$
$$\tau_{ge} \frac{dg_e}{dt} = -g_e$$
$$\tau_{gi} \frac{dg_i}{dt} = -g_i$$

Variable firing threshold

$$\tau_\vartheta \frac{d\vartheta}{dt} = -\vartheta$$

Reset equation

$$u > \vartheta - \vartheta_{offset} + \vartheta_0$$
$$timer > t_r$$

Firing update

$$u \leftarrow u_{reset}$$
$$\vartheta \leftarrow \vartheta + \vartheta_{update}$$
$$timer \leftarrow 0$$

STDP

$$\tau_{pre} \frac{dpre}{dt} = -pre$$
$$\tau_{post1} \frac{dpost1}{dt} = -post1$$
$$\tau_{post2} \frac{dpost2}{dt} = -post2$$

Presynaptic update

$$pre \leftarrow pre_0$$
$$w \leftarrow w - \alpha_{pre} \cdot post1$$

Postsynaptic update

$$post2_{prev} \leftarrow post2$$
$$w \leftarrow w + \alpha_{post} \cdot pre \cdot post2_{prev}$$
$$post1 \leftarrow post1_0$$
$$post2 \leftarrow post2_0$$

Hyper-parameters

$$u_0 = -65\,mV$$
$$u_{ge} = 0\,mV$$
$$u_{gi} = -100\,mV$$
$$\tau_u = 100\,ms$$
$$\tau_{ge} = 1\,ms$$
$$\tau_{gi} = 2\,ms$$
$$\tau_\vartheta(100 neurons) = 10^6$$
$$\tau_\vartheta(400 neurons) = 5 \cdot 10^6$$
$$\tau_\vartheta(1600 neurons) = 10^8$$
$$\vartheta_{offset} = 20\,mV$$
$$\vartheta_0 = 20\,mV$$
$$t_r = 5\,ms$$
$$u_reset = -52\,mV$$
$$\vartheta_{update} = 0.05\,mV$$
$$\tau_{pre} = 20\,ms$$
$$\tau_{post1} = 20\,ms$$
$$\tau_{post2} = 40\,ms$$
$$\alpha_{pre} = 0.0001$$
$$\alpha_{post} = 0.1$$
$$pre_0 = 1$$
$$post_1 = 1$$
$$post_2 = 1$$
$$fi_{min} = 0$$
$$fi_{max} = 64$$
$$\lambda = 78\,mv$$
$$T = 350\,ms$$
$$w^{in} = 17.0$$
